# Canada's *Assisted Human Reproduction Act:* Pragmatic Reforms in Support of Research

**DOI:** 10.3389/fmed.2019.00157

**Published:** 2019-07-10

**Authors:** Tania Bubela, Erika Kleiderman, Zubin Master, Ubaka Ogbogu, Vardit Ravitsky, Amy Zarzeczny, Bartha Maria Knoppers

**Affiliations:** ^1^Faculty of Health Sciences, Simon Fraser University, Burnaby, BC, Canada; ^2^Centre of Genomics and Policy, Department of Human Genetics, McGill University, Montreal, QC, Canada; ^3^Mayo Clinic Center for Regenerative Medicine, Rochester, MN, United States; ^4^Biomedical Ethics Research Program, Mayo Clinic, Rochester, MN, United States; ^5^Faculties of Law, Pharmacy and Pharmaceutical Sciences, University of Alberta, Edmonton, AB, Canada; ^6^Department of Social and Preventive Medicine, School of Public Health, University of Montreal, Montreal, QC, Canada; ^7^Johnson Shoyama Graduate School of Public Policy, University of Regina, Regina, SK, Canada

**Keywords:** assisted reproductive technologies, regulation, criminal law, constitutional law, *in vitro* research, mitochondrial replacement therapy, germline gene editing, Canada

## Abstract

Canada's *Assisted Human Reproduction Act* is long overdue for Parliamentary review. We argue that the current regulation of research using human reproductive materials is not proportionate, not responsive to the uncertain threats posed to human and environmental health and safety, and is not considerate of diverse values in a democratic society. We propose tailored regulatory carve-outs for *in vitro* research for currently prohibited activities, such as gene editing, and for the exercise of Ministerial Discretion for access by Canadians to experimental *in vivo* interventions that are currently prohibited, such as mitochondrial replacement therapy. Our recommendations are bounded by constitutional constraints that recognize political and practical challenges in keeping oversight of this research under Federal jurisdiction, whether conducted in academic or private sectors. The proposed nuanced regulatory scheme should be overseen by a new national Agency, modeled on a blend of the Canadian Stem Cell Oversight Committee and Assisted Human Reproduction Canada.

## Introduction

Robust regulation of novel health biotechnologies in morally contentious domains is central to the ethical conduct of research and clinical applications. Such regulation ought to be proportionate, responsive to the uncertain threats posed to human and environmental health and safety, and considerate of diverse values in a democratic society. Proportionality implies an evidence-informed weighing of the risks and benefits, which in the domain of assisted reproductive technologies (ARTs), may bring communities into conflict. Responsiveness implies that a regulatory scheme should not be static, instead employing principled mechanisms to respond to emerging evidence of benefits, threats, and evolving societal values. Uncertainty is addressed over time through the development of an evidence-base on which regulatory frameworks are premised; the evidence base fits the benefit-harm-uncertainty profile of the assets or products the frameworks are designed to oversee.

ARTs raise rights-related questions with human dignity at their core. How should a regulatory scheme balance the respective rights and considerations outlined in the preamble of the 2004 *Assisted Human Reproduction Act (AHRA)* (1)? These include the interests of researchers and clinicians to advance health/medical innovation, the rights of patients to equitable access to health interventions that have proven to be safe and effective, the rights of women and children to be protected from exploitative practices, as well as consideration of religious freedoms and the “integrity of the human genome.” The question of a balanced regulatory scheme was central to discussions at a multi-stakeholder workshop, convened in Ottawa on December 11, 2018 to deliberate on the *Consensus Statement: Gene Editing, Genetic Testing, and Reproductive Medicine in Canada* (2). The Consensus Statement derived from a consultation process, supported by the Stem Cell Network, on activities that are currently prohibited by the *AHRA* (3–6).

The aim of the workshop was to inform proportionate and responsive regulation of research using human reproductive materials and clinical application of ARTs. Workshop participants included 20 experts in law, ethics, science, and reproductive medicine, as well as five representatives and observers from departments and agencies of the Canadian government. Workshop discussions focused on reforms to the *AHRA* and nuanced regulatory mechanisms that could distinguish between *in vitro* pre-clinical research, clinical research and clinical practice. Here, we make pragmatic recommendations to address the regulation of ARTs, within the constraints of Canada's legal and political framework. Our discussion focuses on pre-clinical research and access by Canadians to ARTs that are currently prohibited by law and accompanied by penal and monetary sanctions. Our discussion does not consider reimbursement of ARTs by Provincial governments or private payers.

In enacting the *AHRA*, the Government of Canada employed its criminal law power to prohibit some areas of research and clinical practice that raised societal concerns in the 1990s and early 2000s. This use of the criminal law power was necessitated by peculiarities in Canada's constitutional division of legislative powers between Federal and Provincial governments. The division of powers constrains options for a regulatory environment that is nationally consistent and covers both academic and private-sector actors. Criminal law is within the legislative purview of the Federal Government, while regulation of research, health care delivery, and regulation of medical professionals generally falls within the ambit of Provincial law-making. For pragmatic reasons, therefore, the issue may not be the use of the criminal law power *per se*, but the lack of an attendant regulatory scheme tailored to research and clinical developments in ARTs. Indeed, the current list of specific prohibitions bans promising avenues of research, while simultaneously allowing some ARTs to escape a similar level of scrutiny, such as pre-implantation genetic diagnosis.

The original intent of the criminal prohibitions contained in the *AHRA* may have been consistent with accepted norms at the time of its enactment, concerns about safety, and a commitment to striking the right balance between promoting science and a precautionary approach to potential risks. However, these controls need to be supplemented by a regulatory scheme that is nuanced and responsive to *current* and *future* developments. This is not to say that the prohibitions are or were immoral or unethical, or that lifting prohibitions on some activities while regulating others would be tantamount to dismissing legitimate concerns. Rather, a move to revisit the prohibitions is consistent with a proportionate, responsive, and considerate approach to regulating science and technologies. It will bring necessary clarity to promising areas of research that have become legally ambiguous in light of emerging techniques. It will provide the public(s) with an assurance that the safety and ethical issues are being seriously attended to within a responsive oversight regime, while providing greater clarity for pre-clinical and clinical researchers, research sponsors, and others with an interest in research outcomes.

That “hard cases make bad law” is a truism. The recent controversy over human germline gene editing in China, therefore, formed a backdrop to the final workshop discussions. In November 2018, Dr. Jiankui He, a biophysicist at the Southern University of Science and Technology in Shenzhen, announced the birth of twin girls whose embryonic genomes he had edited using CRISPR/Cas9 to confer resistance to HIV by removing the *CCR5* gene (7). The claim has since been confirmed; the independently wealthy Dr. He had funded the research himself and allegedly side-stepped ethics oversight at his institution (8). At the time of writing, He is under investigation and house arrest, facing allegations of corruption, bribery and contravening research guidelines that ban genetically modified embryos from being implanted into a human in China (9). These research guidelines are enforceable and any violations can lead to penalties and sanctions (10, 11). The global scientific community has condemned Dr. He's actions, and he has been fired by his research institution (12, 13). Given that He faces both monetary penalties and a prison term, it is unlikely that greater criminal sanctions will deter rogue actors (14). While some have called for a moratorium on germline gene editing, in other circles this event has sparked a broader debate on proportionate regulation that enables responsible progress in this field, based on transparency and robust pre-clinical research and a focus on clear medical need (15–17). In response, the Chinese government has issued new draft regulations that would tighten oversight for “high-risk biomedical technologies,” which includes gene editing (18). Canadian regulatory reform discussions should take notice of this event, but not be overly influenced by it (19).

Elsewhere, the authors have argued for a distributed governance model for ARTs and research involving human embryos and reproductive material that engages Federal and Provincial ethics oversight, informed by the 2014 *Tri-Council Policy Statement on Ethical Conduct for Research Involving Humans* (TCPS2) (20), and provincial professional regulation (5). While replacing the *AHRA* with a new legal framework might be the preferred long-term approach to addressing current issues, we recognize the associated political and practical challenges. Therefore, the consensus of the present workshop was to outline what may be a more feasible way forward in the short term. We focused our discussion on reforms to the *AHRA* concomitant with the development of regulations and a regulatory agency that would enable unified Federal oversight of research, whether conducted in academic or private sectors. This latter point is significant, because the nationally harmonizing effect of the TCPS2 over ethical conduct of research involving human reproductive materials is limited to institutions that are eligible to receive Tri-Council research funding. Private sector entities, therefore, unless they collaborate with Tri-Council eligible institutions or voluntarily adopt the Policy, do not necessarily fall under the auspices of the TCPS2.

Our focus is on proposing tailored regulatory carve-outs for *in vitro* research for currently prohibited activities and for the exercise of Ministerial Discretion by the Minister of Health for access by Canadians to experimental *in vivo* interventions that are currently prohibited under the *AHRA* [e.g., mitochondrial replacement therapy (MRT)] (4, 21). The latter recommendation is a short-term compromise while researchers determine whether such interventions are safe and effective; thereby demonstrating that a criminal ban is no longer justified, unless social consensus still considers such an intervention as morally reprehensible. Many have argued that such social consensus does not presently exist for many ARTs, even if it may have existed in the period leading up to the enactment of the *AHRA* (22, 23). Justifying our recommendations requires a foray into the checkered history of the *AHRA*, as well as Canadian constitutional and administrative law.

## The Criminal Law Power and the *Assisted Human Reproduction Act*

The *AHRA* is long overdue for its Parliamentary Review, mandated within 3 years of the establishment of the Assisted Human Reproduction Agency (the Agency) that was slated to oversee the operations of the *AHRA* (s. 741–745) (1). The Agency was created shortly after the *AHRA* came into force. However, it was disbanded shortly thereafter when the Supreme Court of Canada (SCC), in a divided decision with three sets of reasons, struck down most of the provisions of the *AHRA* after a constitutional challenge over legislative jurisdiction from the Province of Quebec (24). The impugned provisions included those that regulated ARTs in fertility clinics; most of these controlled activities were found to be an unconstitutional incursion into Provincial jurisdiction over health services. However, a majority of justices upheld sections 8, 9, and 12 that regulated surrogacy and donation of human reproductive material as a valid exercise of criminal law. Health Canada has recently held a consultation on regulatory reforms to aspects of these provisions, including the safety of sperm and ova, reimbursement to women for activities associated with surrogacy, and related administration and enforcement (25). This signals the willingness of Health Canada to open regulatory discussions after the only known criminal charges were brought against the operator of a fertility service and her company for remunerating surrogacy and egg donation. The accused plead guilty, so there were no written reasons to clarify the scope of payments allowable for these services (26).

Not challenged as being within Federal jurisdiction were the criminal prohibitions in sections 5–7. Section 5 details prohibited research and clinical activities, including human cloning, creation of embryos for research purposes, human embryonic research beyond 14 days of development, sex selection for non-medical reasons, human genome alterations capable of being transmitted to future generations, transplantation of non-human reproductive material into a human and vice versa, creation of chimera, and creation of human-non-human hybrids for the purposes of reproduction or transplantation into a human. Section 6 prohibits some activities related to surrogacy and section 7 prohibits the purchase and sale of reproductive material and its use without appropriate consent.

We focus our discussion on the prohibitions in section 5 that were not challenged and whose constitutional validity on jurisdictional grounds is therefore presumed based on tangential consideration by the SCC justices, known as *obiter*. To our knowledge, no legal action has ever been taken to enforce the s. 5 prohibitions. Of particular interest is the judicial reasoning on the scope and relevance of the criminal law in this context. Valid criminal law takes the form of a prohibition backed by a penalty for a legitimate criminal law purpose, including “[p]ublic peace, order, security, health, morality” (p. 50) (27). In considering the legitimacy of the section 5 prohibitions, the three sets of judgments refer to “conduct that is reprehensible” (para. 26) and “reflecting pressing moral concerns” (para. 79) (24). Indeed, “the dominant effect of the prohibitory and administrative provisions is to create a regime that will prevent or punish practices that may offend moral values, give rise to serious public health problems, and threaten the security of donors, donees, and persons not yet born” (para. 32) (24).

On the subject of morality, the Chief Justice stated:

[a]ssisted reproduction raises weighty moral concerns. The creation of human life and the processes by which it is altered and extinguished, as well as the impact this may have on affected parties, lie at the heart of morality. Parliament has a strong interest in ensuring that basic moral standards govern the creation and destruction of life, as well as their impact on persons like donors and mothers. Taken as a whole, the Act seeks to avert serious damage to the fabric of our society by prohibiting practices that tend to devalue human life and degrade participants. This is a valid criminal law purpose, grounded in issues that our society considers to be of fundamental importance (para. 61) (24).

It is not necessary for there to be societal consensus on the morality of specific acts. Rather, “[p]arliament need only have a reasonable basis to expect that its legislation will address a moral concern of fundamental importance, even if hard evidence is unavailable on some points because ‘the jury is still out”' (para. 50) (24).

## Regulatory Approaches Under the Criminal Law

The *AHRA Reference* decision suggests that the remaining statutory prohibitions are valid and likely to survive a challenge on jurisdictional grounds. However, the decision also suggests that valid criminal law is capable of supporting a nuanced regulatory scheme that permits “flexibility,” which is “vital in a field of evolving technologies.” Regulations are simpler to reform and therefore more nimble statutory instruments than Acts, which are subject to full Parliamentary review of proposed reforms. Most Acts are operationalized through regulations, which though comprising hard law in the form of subordinate legislation, are made or enacted by the responsible Minister rather than Parliament. Indeed, Health Canada derives its power to regulate drugs, medical devices, and clinical research from the criminal law and protection of public safety (28, 29). For example, the prohibition of the sale of drugs states that “[n]o person shall sell any drug that (a) was manufactured, prepared, preserved, packaged, or stored under unsanitary conditions; or (b) is adulterated” (s. 8) (30). Its comprehensive approach to regulation is done under the umbrella of the *Food and Drugs Act* (30) and subordinate regulations, such as the *Food and Drugs Regulations* (31), *Cannabis Regulations* (32)*, Medical Devices Regulations* (33)*, Processing and Distribution of Semen for Assisted Conception Regulations* (34)*, Safety of Human Cells, Tissues, and Organs for Transplantation Regulations* (35), and *Blood Regulations* (36). These statutory instruments collectively set out a fulsome regulatory scheme that permits some activities, while imposing penalties for non-compliance.

In numerous arenas, therefore, we have working examples of nuanced regulatory schemes, backed by criminal law powers, capable of protecting public health and safety, without overly restricting health innovation. Working on the assumption that there is limited political will to repeal the *AHRA*, the consensus of the workshop was to recommend regulatory carve-outs to advance research in the domain of ARTs or using human reproductive materials, without compromising human health and safety. Such an approach has recently been taken, for example, to enable medical assistance in dying (MAiD) in Canada. MAiD had been subject to the criminal prohibition against counseling or aiding suicide and procuring consent to death. Enabling MAiD required amending the *Criminal Code of Canada* to allow a “carve-out” for patients who meet specified criteria to receive assistance with dying from physicians or nurse practitioners (s. 241) (37). The implementation of MAiD as a health service therefore falls to a distributed governance model between the Federal government and the Provinces under whose jurisdiction falls the delivery of health care through their health authorities as well as the regulation of health professionals through professional associations. A similar regulatory carve-out from criminal prohibitions existed for medicinal marijuana, prior to its legalization in Canada [s. 55(1)] (38). These examples represent the evolution of criminal law as societal values and views of what comprises reprehensible conduct shift over time.

What then would a regulatory carve-out under the statutory prohibitions of the *AHRA* include that reflects current knowledge and social values? In our view, *in vitro* pre-clinical research, currently prohibited under the *AHRA* should be permitted, subject to the 14-day rule for research involving human embryos (see Table 1). Such a regulatory carve-out would protect the safety and interests of Canadians, especially women, and allow Canadians the opportunity to benefit from advances in knowledge in the fields of genomic and regenerative medicine (77, 78). Enabling such research is in the interests of Canadians who carry known mutations for rare diseases, which may benefit from gene editing approaches; currently only pre-implantation genetic diagnosis of carrier embryos is available.

**Table 1:** Activities that would be enabled under a regulatory carve-out for *in vitro* research.

**Prohibited research activity**	**Examples of *in vitro* research that would be enabled under a regulatory carve-out**	**Examples of jurisdictions in which such *in vitro* research is permitted**
**5(1)(a)** create a human clone^§^ by using any technique, or transplant a human clone into a human being or into any non-human life form or artificial device	Somatic Cell Nuclear Transfer (SCNT) is a technique in which the nucleus of a somatic cell (from almost anywhere in the body) is transferred into an oocyte (egg) that has had the nucleus removed. The egg, which then carries a near genetic copy of the source material can be “stimulated” to divide (39–41). The *AHRA* does not distinguish between cloning for reproductive purposes and using SCNT for research or therapeutic purposes. The latter creates a “clone” for the purpose of harvesting stem cells that might be used in treating a disease or disability in the person from whom the “clone” was generated. In other words, it creates “personalized” stem cell lines” (42).	Typically permitted in jurisdictions that allow the creation of embryos for research purposes. This technique for generating stem cells has become largely redundant due to the discovery of induced pluripotent stem cells (42). For example, **China** (*Ethics Guiding Principles for Human Embryonic Stem Cell Research*, 2004); **Israel** (*Report of the Bioethics Advisory Committee of the Israel Academy of Sciences and Humanities: The Use of Embryonic Stem Cells for Therapeutic Research*, 2001); **Singapore** (*Ethics Guidelines for Human Biomedical Research, Bioethics Advisory Committee of Singapore*); the **United Kingdom** (*Human Fertilisation and Embryology Act, 1990*); and **some states in the United States** (California, Massachusetts, New Jersey) permit the use of SCNT for research or therapeutic purposes. Yet, the United States bans the use of federal funding for research uses of SCNT.
**5(1)(b)** create an *in vitro* embryo for any purpose other than creating a human being or improving or providing instruction in assisted reproduction procedures	Leftover embryos from IVF clinics are generally already 5 days old. Access to earlier embryos enables research into the events surrounding fertilization; early embryonic development and epigenetic reprogramming (i.e., origins of adult diseases); better understanding of the molecular events of early human embryos (e.g., activation of the embryonic genome); observing human egg and sperm interaction/signaling; and improving quality assessment of gametes (43, 44). In addition, uncertainty exists as to the permissibility of the creation of structures that resemble embryos (45, 46) known as synthetic human entities with embryo-like features (47, 48). These structures are a valuable research tool for understanding early embryonic development and developmental disorders (48, 49).	15 countries permit the creation of embryos for research purposes, at least to some extent (50). For example, **Australia**, **Belgium**, **Canada**, **China**, **Denmark**, **India**, **Israel**, **Japan**, **Singapore**, **South Africa**, **South Korea**, **Spain**, **Sweden**, the **United Kingdom**, and the **United States (not all states)**.
**5(1)(f)** alter the genome of a cell of a human being or *in vitro* embryo such that the alteration is capable of being transmitted to descendants	Research into **gene editing** to correct known genetic mutations, for example, Duchenne Muscular Dystrophy, or ß-thalassemia (51). Reports generated by the National Academies of Sciences, Engineering, and Medicine (2017), as well as the Nuffield Council on Bioethics (2018) state that human germline modification is not unacceptable in and of itself; as such its use may become morally acceptable in time (52, 53). Both reports envision the possibility for clinical trials, one day, under specific circumstances (e.g., serious, life-threatening diseases), provided stringent criteria are met and rigorous oversight is in place (e.g., licensing body). To date, such *in vitro* research includes: **China** Research using germline modification on non-viable human embryos to study ß-thalassemia (54); Research using germline modification on non-viable human embryos to study HIV (55). **United Kingdom** A license granted by the HFEA in February 2016 for the use of germline modification on viable human embryos to better understand implantation failure and miscarriage (56, 57). **Sweden** Research using germline modification on viable human embryos to better understand implantation failure and miscarriage (60). **United States** Research using germline modification on viable human embryos to correct a heritable heart condition and to better understand the safety and efficacy issues surrounding CRISPR/Cas9 (61, 62). *Note that all embryos were destroyed within the 14-day window. Research into **mitochondrial replacement therapy** (4). To date, research on the safety and efficacy of the technology has been conducted in both animal models (mice and non-human primates) and human oocytes (63–65); yet clinical trials are not permitted. Research to better understand the maternal factors that prevent embryo development and trigger embryo arrest (48). This can be done by providing healthy ooplasm to support proper nuclear activation and reprogramming (66).	Several countries draw a clear distinction between the application of human germline modification in a research vs. clinical context, permitting the former provided regulatory approval has been received and stringent criteria met. For example, **Belgium** (*Embryo Research Law*, 2003); **Singapore** (*Human Cloning and Other Prohibited Practices Act (Human Cloning Act)*, 2004; *Ethical, Legal, and Social Issues in Human Stem Cell Research, Reproductive and Therapeutic Cloning*, 2002); and the **United Kingdom** (*Human Fertilisation and Embryology Act 1990*). The **United Kingdom** was also the first country to legalize mitochondrial replacement therapy (*The Human Fertilisation and Embryology (Mitochondrial Donation) Regulations 2015*)—a license from the HFEA is required (58, 59).
**5(1)(i)** create a chimera^†^, or transplant a chimera into either a human being or a non-human life form	Research into the development of human organs and the developmental origins of human disease (67, 68). Research to better understand the lineage of human primordial germ cells and how they specify. Such research would be facilitated via chimeric studies using human embryonic stem cells that are implanted into the embryo of another species (e.g., a pig or cow) so as to study the stimulating pathways and gain a better understanding of early embryo development (69, 70). Yet, this would involve studying the chimeric embryos beyond the 14-day window, which may fall into a gray area with regards to the *AHRA*. As well, the costs associated with the source of gametes makes them financially inaccessible to federally funded researchers. These costs could be decreased by improving our understanding of *in vitro* gametogenesis (i.e., creating gametes using stem cell technology) (71). Currently being tested in mouse models, it has become clear that there are certain differences that need to be further understood within the human context (72).	Several countries permit the creation of chimeras for research purposes. For example: **Japan** used to allow human-animal chimera research only up until the 14th day (or the appearance of a primitive streak). In March 2019, however, the Ministry of Education, Culture, Sports, Science, and Technology revised the *Guidelines for the Handling of Specified Embryos* to lift the 14-day limit (73). **Germany** forbids combining human embryos with animal cells, but not the introduction of human cells into an animal embryo (74). The **United Kingdom** does not prohibit the creation and use of admixed embryos so long as “the Authority is satisfied that any proposed use of embryos or human admixed embryos is necessary for the purposes of the research” (*Human Fertilisation and Embryology Act 1990* (as amended in 2008) at Schedule 2, s. 3(3) and s. 3(5)). The **United States** might have a generally permissive policy. In August 2016, the NIH issued a draft policy in which it sought to lift a moratorium on the federal funding of research involving the introduction of human pluripotent cells into vertebrate embryos (75). **France** forbids the creation of chimeric human embryos (*Code de la santé publique*, 2000, L. 2151-2), but is unclear as to whether adding human cells to animal embryos is permitted (76).

§ ***Chimera** “means (a) an embryo into which a cell of any non-human life form has been introduced; or (b) an embryo that consists of cells of more than one embryo, fetus or human being” (s. 3 AHRA)*.

† ***Human clone** “means an embryo that, as a result of the manipulation of human reproductive material or an in vitro embryo, contains a diploid set of chromosomes obtained from a single—living or deceased—human being, fetus or embryo” (s. 3 AHRA)*.

Under our proposal, ART research would be overseen by a regulatory agency, modeled on a blend of the national Stem Cell Oversight Committee (SCOC) and Assisted Human Reproduction Canada. Currently, the SCOC oversees research involving pluripotent stem cells, including human embryonic stem cells. The SCOC is administered within the Canadian Institutes of Health Research (CIHR) and oversees compliance of researchers who, by nature of their work affiliation, are required to comply with TCPS2 (5). The SCOC also oversees compliance with the *AHRA*, whose statutory prohibitions are reflected in TCPS2. For its part, Assisted Human Reproduction Canada was a federal regulatory agency (2006–2013) whose mandate was to “administer and enforce the [*AHRA*] and related regulations in order to protect and promote the health, safety, dignity and rights of Canadians who use or are born of assisted reproductive technologies” (p. 3) (79).

A new Agency with an expanded mandate under the *AHRA* and any new regulations would necessarily be charged with oversight of a broader range of research activities by all Canadian researchers, whether subject to TCPS2 or not (see Table 2). Even in its reduced form following the Constitutional challenge, the *AHRA* retains provisions for an oversight Agency, regulation making power, and general powers for administration and enforcement of the criminal prohibitions. Such an Agency could increase public confidence in its oversight through multi-stakeholder representation, including ethicists and members of the public. In this way, the Agency would follow best practices for citizen participation in regulation and deliberative process (81). An Agency with this mandate would be more likely to be constituted in the current climate, because of the issues at stake and greater confidence in the constitutionality of the remaining provisions of the *AHRA*.

**Table 2:** Contours of a new regulatory agency to oversee research using ART.

**Current Stem Cell Oversight Committee (SCOC) 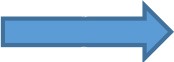 **	**Proposed new regulatory agency [modeled on a blend of SCOC and Assisted Human Reproduction Canada (79)]**	**Similar regulatory agencies in other countries**
Standing committee of the CIHR Governing Council (80). **Composition** Chair + 9 additional members (minimum), selected by the CIHR Governing Council. ° One *ex officio* member of the Interagency Advisory Panel on Research Ethics; ° President of CIHR is an *ex officio* member. Expert members: stem cell biology and therapeutics, developmental biology or embryology; health care (e.g., a professional specializing in reproductive medicine); ethics; law; and social sciences. Other members: voluntary health sector, public and patient groups, international stem cell research policy. **Mandate** Oversight and review of applications for research on human pluripotent stem cells—including embryonic stem cells, induced pluripotent stem cells and embryonic germ cells—to ensure conformity with the TCPS 2. ° National, complementary review to local REB review. Advisory role regarding scientific, ethical, legal and social implications of human stem cell research and its possible clinical applications.	**Governance** Departmental Corporation, supported by Health Canada, reporting to the Minister of Health. Governed by a Board of Directors, including the Chair, and the President of the new Agency. Board Members appointed by the Governor in Council. Membership similar to SCOC, but appointed by the Governor in Council, including public member(s). **Mandate** *Regulatory Compliance:* promote compliance and enforce the *AHRA* and associated regulations related to the prohibitions and regulated activities, within a sound ethical framework; *Knowledge Exchange:* Provide information to the public and to the professions respecting those activities that fall within the mandate of the *AHRA* and related matters; Monitor and evaluate developments within Canada and internationally in those activities that fall within the mandate of the *AHRA* and related matters; Consult persons and organizations within Canada and internationally; and, *Internal Services:* Provide advice to the Minister on those activities that fall within the mandate of the *AHRA* and related matters. **Strategic outcome** Protection and promotion of health and safety of Canadians in relation to those activities that fall within the mandate of the *AHRA*, within a sound ethical framework.	Human Fertilisation and Embryology Authority (United Kingdom) Embryo Research Licensing Committee (Australia) Federal commission for medical and scientific research on embryos *in vitro* (Belgium) Biomedicine Agency (Agence de la biomédecine) (France) Ministry of Technology and Ministry of Health (Japan) Bioethics Advisory Committee (Singapore)

While most of the research activities outlined in Table 1 are not yet ready for clinical application, diagnostic, and therapeutic interventions derived from such research will likely become available in the future. Some, such as MRT, have already been approved for clinical use in other countries, such as the United Kingdom (82). The United Kingdom's Human Fertilization and Embryology Authority (HFEA) has licensed one clinic and has approved the first application for the use of mitochondrial donation to treat patients (58). The clinic has been licensed to perform the technique but must still apply to the HFEA to treat individual patients (83). The latest statistics suggest that 15 babies have been born via MRT in five countries: Ukraine, United States (with MRT performed in Mexico), Israel, Sweden, and Greece (84, 85).

In the Canadian context, it is an open question whether prospective parents could be prosecuted under *AHRA* if they availed themselves of MRT in a country where the practice is permitted (21). This was the issue in the successful constitutional challenge of the prohibition on assisted suicide (86). The daughter of a Kay Carter, a woman with spinal stenosis, was concerned about being prosecuted upon her return to Canada after assisting her mother to end her life at the Dignitas clinic in Switzerland. She successfully argued, under section 7 of the *Canadian Charter of Rights and Freedoms* (87) that the prohibition was overly broad and therefore unjustifiably infringed her rights to life, liberty, and security of the person. This avenue of a *Charter* challenge is open to parents who wish to access MRT as well as to researchers, whose liberty is threatened by the penal sanctions in the *AHRA*. Indeed, the SCC in considering the constitutionality of the prohibited activities under jurisdictional grounds, left the door open to such a *Charter* challenge.

Accordingly, is there an available mechanism that might enable controlled access to currently experimental ARTs as their safety and efficacy profiles are ascertained? One option might be to enable a Ministerial Exemption on a case-by-case basis from the operation of the *AHRA*, following a transparent decision-making process and subject to judicial review. Such a scheme exists, for example, to exempt health practitioners, and other staff of supervised injection facilities from the operation of the *Controlled Drugs and Substances Act* (*CDSA*) (38). Section 56(1) of the *CDSA*. This section enables the Minister to exempt a person from any of the provisions of the *CDSA* “if, in the opinion of the Minister, the exemption is necessary for a medical or scientific purpose or is otherwise in the public interest.” The *CDSA* and Regulations then specify the conditions and processes for the exemption, and the Ministerial decision is subject to judicial review, which limits the risk that the decision will be unfair or arbitrary (88).

Recommendations for regulatory reform and ministerial discretion, however, are only practical if they can be implemented. The question is whether the regulatory approaches we have outlined are currently permitted under the *AHRA*. It could be argued that the general provision, under section 65(1) that “[t]he Governor in Council may make regulations for carrying into effect the purposes and provisions of this Act” is broad enough to create regulatory carve-outs for research purposes. However, the regulatory powers specified in this section refer to repealed sections, or sections other than the statutory prohibitions in section 5. Specifically, regulation-making authority currently rests with the Federal Cabinet (Governor in Council) and covers *only* the controlled aspects of the *AHRA* (section 8—“use of reproductive material without consent” and section 12—“reimbursement of expenditures”). The proposed regulatory carve-outs, would therefore, likely require amendments to the *AHRA*. The *Criminal Code* (37) similarly was amended to create the regulatory carve-outs for MAiD, including powers to make regulations. However, with broader regulation-making powers, the regulatory carve-out for medicinal marijuana from the *CDSA* was made under the *Access to Cannabis for Medical Purposes Regulations* (89), which did not require amendments to its Act.

However, while creating regulations may require a less onerous process than amending an Act, that distinction may be less pronounced with the *AHRA*. Under section 66, the *AHRA* takes the unusual step of requiring regulations made under section 65 to be laid before each House of Parliament by the Minister of Health. That process requires a review of the proposed regulation and a report on the findings to the House by the appropriate committee of each house, such as the Standing Committee on Health. These extra steps were required to enhance transparency and public confidence in the operation of the *AHRA*. Notably, there is no provision for ministerial discretion, which would require reforms to the *AHRA* itself.

## Conclusion

In conclusion, the *AHRA* is long overdue for Parliamentary review and requires updates to reflect societal changes and scientific progress. Here, we have argued for a proportionate, responsive, and considerate regulatory regime for research using human reproductive materials that are currently prohibited in Canada. Our recommendations are bounded by constitutional constraints that recognize political and practical challenges in keeping oversight of this research under Federal jurisdiction, whether conducted in academic or private sectors. In our view, a nuanced regulatory scheme, overseen by a national Agency, could enable some currently prohibited *in vitro* research activities, while protecting the safety and interests of Canadians, especially women. Recommended reforms include a regulatory carve-out for some *in vitro* research activities and exercise of ministerial discretion for promising clinical research, for example, to enable MRT. In the absence of reform or a fulsome societal debate for Parliament to replace the *AHRA* with a more workable framework, the only recourse for researchers and patients who might benefit, will be through *Charter* challenges to the remaining provisions of the *AHRA*. That possible avenue was acknowledged by the SCC in the *AHRA* reference case (24), but such legal actions are fraught with difficulties and obstacles that researchers and/or patients are unlikely to undertake.

## Author Contributions

TB, EK, UO, VR, AZ, and BK contributed to the conception and design of the study. TB and EK wrote the first draft of the manuscript. All authors contributed to manuscript revision and acquisition of data, read, and approved the submitted version.

### Conflict of Interest Statement

The authors declare that the research was conducted in the absence of any commercial or financial relationships that could be construed as a potential conflict of interest.
